# Epigenetic regulators in cancer therapy and progression

**DOI:** 10.1038/s41698-025-01003-7

**Published:** 2025-06-28

**Authors:** Hany E. Marei

**Affiliations:** Department of Cytology and Histology, Faculty of Veterinary Medicine, Mansoura University, Mansoura, Egypt

**Keywords:** Cancer, Oncology

## Abstract

Heritable gene activity variations that do not alter the underlying DNA sequence are known as epigenetic modifications. Mutations that disrupt genome structure and function are key drivers of oncogenesis. In addition to genetic mutations that cause direct disruptions in the DNA sequence, epigenetic changes can affect gene expression, which helps the development of cancerous traits. Changes in DNA methylation and histone mark patterns are the main drivers of these epigenetic modifications, as they encourage the growth and spread of tumors. In this review, we explore the substantial implications of epigenetic control on tumor genesis, metastatic behaviour, metabolic pathways, and the tumor microenvironment, delving into the intricacies of this intricate regulation. We pay special attention to the dysregulation at every stage of epigenetic modulation, which includes, but is not limited to, abnormalities in the enzymes that modify histones and methylate DNA, subunit loss or fusions in chromatin remodeling complexes, and disruptions in higher-order chromatin structure. We also highlight the development of inhibitors of medications targeted at epigenetic enzymes and summarize the abnormal roles of enzymes in DNA methylation, histone acetylation, and histone methylation during tumour progression.

## Introduction

The notion that changes in cellular phenotype are passed across generations, regardless of changes in DNA sequence, was named epigenetics when it was first described^1^. Conrad Waddington coined the word “epigenetics” in 1942 to explain the relationship between genotype and phenotype^2^. Classical genetics has long held that DNA sequences of DNA determine cell phenotypes, a belief that dates back to the discovery of DNA and the double helix structure. In cells, DNA is bundled into chromatin, with nucleosomes serving as the basic repeating unit. An octamer of four core histones (H2A, H2B, H3, and H4) is encircled by a 147 base-pair (bp) length of DNA. The usual description of cancer is that it is a collection of several diseases, each caused by a different mutational mechanism and requiring a different course of treatment. There are similarities in the epigenetic makeup of cancer types, especially with relation to tumor heterogeneity and medication resistance, even though the cancer types differ in terms of the tissue of origin and related mutation spectrum^3^. This common epigenetic environment plays a significant role in phenotypic plasticity, which is essential for intricate changes like increased metastasis and cell proliferation^4^. It has long been known that the diversity and adaptability of cells are essential components driving the development of cancer. Coordinated gene expression programs in these cells often result in complex and diverse behaviors. These programs show a clear divergence from those that defined the initial tissue phenotypes, indicating a more intricate level of cancer biology. Therefore, whereas genetic changes may predispose cells to novel phenotypic states, they do not solely control the establishment of these states or their development^5^. The elements involved in different modification patterns can be divided into three roles: “writer,” “reader,” and “eraser.” The “writers” and “erasers” refer to enzymes that transfer or remove chemical groups to or from DNA or histones, respectively. “Readers” are proteins that can recognize the modified DNA or histones. Generally speaking, epigenetic events involve DNA methylation, histone modification, the readout of these modifications, chromatin remodeling, and the effects of noncoding RNA. High-throughput technologies have significantly improved and expanded our knowledge of epigenetic pathways causing cancer. This progress has revealed a variety of epigenetic markers unique to cancer that may prove to be extremely important as biomarkers for diagnosis, prognosis, and response to treatment. Moreover, epigenetic machinery presents a strong case for therapeutic intervention because of its intrinsic reversibility, in contrast to the irreversible nature of genetic mutations. At the forefront of contemporary drug development research is this element. The epigenome works with other regulatory factors, such as transcription factors and noncoding RNAs, to control the expression or repression of the genome to coordinate various biological activities. Extracellular stimuli and cellular signaling pathways can potentially have an impact on epigenetics. These effects are both transient and persistent. A deeper comprehension of both normal and aberrant epigenetic processes can aid in the understanding of etiology and possible therapeutic approaches for a variety of diseases, including cancer, given the significance of epigenetics in affecting cell functions.

Cancer has a complex etiology that includes both genetic and environmental factors. It is typically possible to identify genetic information modification in cancer cells. Epigenome dysregulation is common in cancer, much like genome instability and mutation. Certain modifications impact the way cells function and have a role in the development of cancer^6^. Nevertheless, the cancerous phenotype can return to normal by using medication or gene therapy to reverse these alterations. The importance of epigenetic changes in oncology is becoming better acknowledged, especially in light of their function as mechanistic factors that promote the development of cancer characteristic features^7^. This realization stems from the discovery of extensive, reversible epigenetic modifications that are strongly impacted by external circumstances and can simultaneously regulate many genes. Surprisingly, these epigenetic changes frequently come before and outweigh hereditary abnormalities. Following a thorough investigation into the genetic and epigenetic changes associated with a variety of pediatric malignancies, a noteworthy fraction with few or no mutations surfaced^8^.

Even if cancer patients in clinical settings have the same staging and grade, their outcomes are completely different. Tumor tissues exhibit diverse histone modification patterns, either across the entire genome or in specific genes, suggesting the presence of epigenetic heterogeneity at the cellular level^9^. Similarly, the use of molecular biomarkers is considered a possible way to stratify patients. It is significant to remember that several epigenetic events work together to cause cancer. For instance, the methylation of DNA CpG islands along with hypoacetylated and hypermethylated histones typically results in the repression of tumor suppressor genes^10^.

Four main epigenetic mechanisms—DNA methylation, histone modification, chromatin remodeling, and higher-order chromatin structure—are methodically outlined in this review. These pathways, which include oncogenesis, metastasis, metabolism, and the tumor microenvironment, are essential for controlling tumor heterogeneity. We highlight the role that epigenetic regulation plays in controlling the variety of gene expressions, which supports both the emergence of new phenotypes and the maintenance of current cellular states. We also assess new and existing therapeutic methods that are based on fast developing understanding of epigenetic mechanisms. Histone acetylation, histone methylation, and DNA methylation were the three areas of epigenetics in cancer that we concentrated on and briefly discussed. Lastly, we provided an overview of the most recent advancements in cancer epigenetic therapy.

## A synopsis of epigenetics

**Fig. 1:**
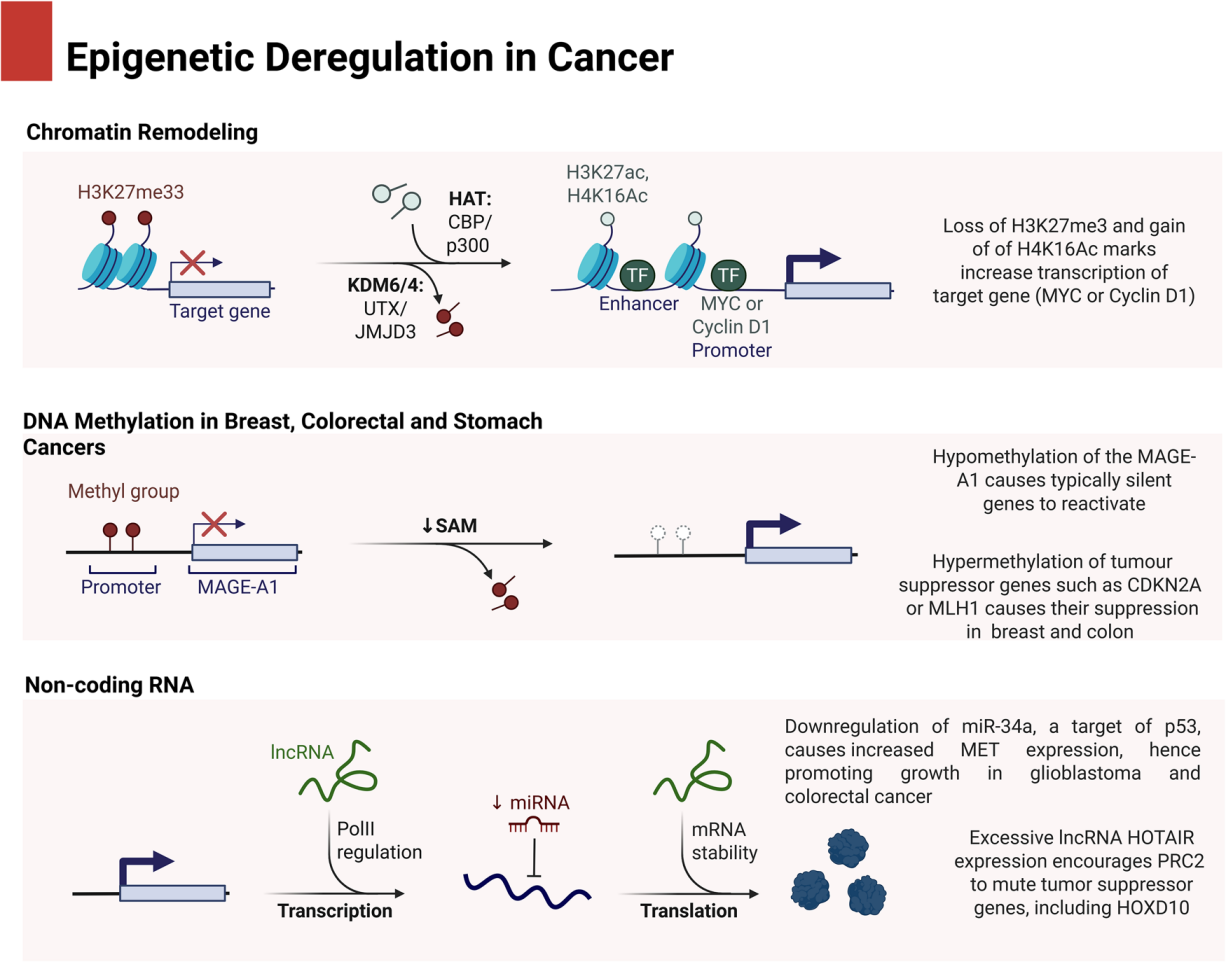
Epigenetic deregulation in cancer. The three main epigenetic processes changed in cancer, shown in this image, are: (1) chromatin remodeling, (2) DNA methylation dysregulation, and (3) non-coding RNA (ncRNA) modification. *Chromatin Remodeling and Histone Modifications*: Changes in histone post-translational modifications (PTMs) characterize cancer-related chromatin remodeling. Histone acetyltransferases (HATs) like CBP/p300 may help to acquire activating histone acetylation marks, such as H3K27ac and H4K16ac, which could then activate oncogenes. In several cancers, including colorectal and breast cancer, increasing H3K27ac near the MYC promoter increases its transcription. Conversely, KDM6A/B (UTX/JMJD3) removes repressive markers such H3K27me3 (trimethylation at lysine 27 of histone H3), causing genes linked to cell development to be derepressed. The decline of H3K27me3 at the CCND1 (Cyclin D1) promoter is an excellent case in point since it drives overexpression in glioblastoma and breast cancer. *DNA Methylation Dysregulation*: Hypomethylation of CpG islands inside promoter regions could lead to abnormal expression of proto-oncogenes. Common in colorectal and stomach cancers, hypomethylation of the MAGE-A1 gene causes typically silent genes to reactivate. Decreased levels of S-adenosylmethionine (SAM), which act as a methyl group donor for DNA methyltransferases (DNMTs), also cause global hypomethylation. On the other hand, hypermethylation of tumor suppressor gene promoters like CDKN2A (p16^INK4a^) or MLH1 causes their transcriptional suppression in cancers, including breast and colon. *Non-coding RNA Dysregulation*: At both the transcriptional and post-transcriptional levels, microRNAs (miRNAs) and long non-coding RNAs (lncRNAs) alter gene expression. Downregulation of miR-34a, a target of p53, causes increased MET expression, hence promoting growth in glioblastoma and colorectal cancer. Similarly, excessive lncRNA HOTAIR expression encourages PRC2 (Polycomb Repressive Complex 2) to mute tumor suppressor genes, including HOXD10, particularly in breast and colorectal cancers. These linked epigenetic mechanisms finally lead to the disturbance of gene expression patterns, hence promoting oncogenesis. Clear identification of specific enzymes—e.g., DNMTs, HATs, HDACs, KDMs—histone modifications—e.g., H3K27ac, H3K27me3, H4K16ac—and gene targets—e.g., MYC, CDKN2A, HOTAIR—in the figure would increase knowledge and mechanical clarity^193^.

Fine-tuning gene expression is greatly aided by epigenetic modifications, which are heritable but reversible changes in gene activity that are independent of changes in DNA sequence. Important biological processes, such as cell differentiation and embryogenesis, are regulated by these changes. Specifically, they play a key role in epigenetic reprogramming, which drives transcriptome heterogeneity in cancer^7^. Originally used to refer to a wide variety of activities outside of conventional gene expression regulation, the term “epigenetics” now more precisely refers to a group of regulatory processes, especially those involving chromatin modifications and DNA methylation. The three main processes of epigenetic dysregulation in cancer: non-coding RNA (ncRNA) participation, DNA methylation dysregulation, and chromatin remodeling. Oncogene overexpression results from chromatin remodeling, which modifies histone markers, including the loss of repressive H4K20me3 and the gain of activating H4K16Ac, mediated by HATs and demethylases like KDM6/4. DNA methylation dysregulation, particularly hypomethylation at CpG sites, is associated with lower S-adenosylmethionine (SAM) levels, resulting in abnormal oncogene activation. Finally, through aberrant gene expression, ncRNA dysregulation—which includes long non-coding RNAs (lncRNAs) and microRNAs (miRNAs)—affects transcription, mRNA stability, and translation, thereby fostering carcinogenesis. When combined, these epigenetic modifications interfere with regular gene regulation, which accelerates the development of cancer **(**Fig. 1**)**.

## DNA methylation

**Fig. 2:**
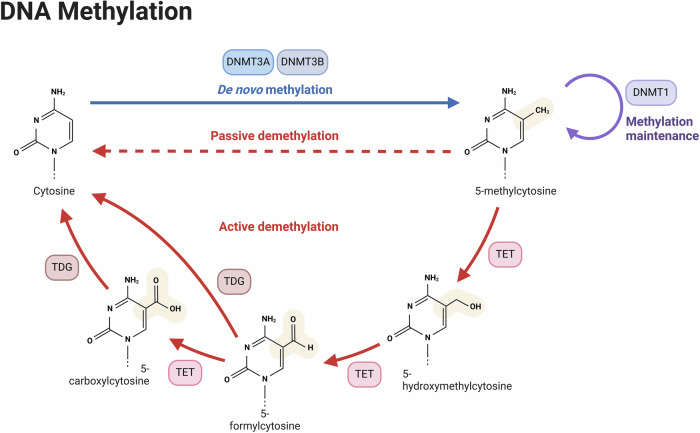
DNA methylation pathway and regulation. The steps involved in DNA methylation, active demethylation, and passive demethylation are depicted in this diagram. DNMT3A and DNMT3B catalyze de novo methylation, which creates new methylation patterns by converting cytosine to 5-methylcytosine. DNMT1 mediates methylation maintenance, which guarantees that 5-methylcytosine is preserved throughout DNA replication. Ten-eleven translocation (TET) enzymes oxidize 5-methylcytosine step-by-step during active demethylation, producing intermediates such 5-hydroxymethylcytosine, 5-formylcytosine, and 5-carboxylcytosine. Thymine DNA glycosylase (TDG) then breaks down these intermediates to restore unmethylated cytosine. When maintenance methylation is unsuccessful, methylation gradually disappears across cell divisions, a process known as passive demethylation. Both gene expression and epigenetic stability are regulated by this dynamic interaction.

The main method that methylation of mammalian DNA happens is by covalently adding a methyl group to the cytosine’s carbon-5 atom in a cytosine-guanine (CpG) dinucleotide. Three DNA methyltransferases (DNMTs) catalyze this enzyme process. Initial DNA methylation marks are created by DNMT3A and DNMT3B, which exhibit similar preference for hemimethylated and unmethylated DNA molecules^11^. According to Peter Laird’s 2003 assessment, the then-recent discoveries on the involvement of DNA methylation in human cancer suggested that a variety of potent DNA methylation-based biomarkers, especially for use in cancer diagnosis, may eventually be developed^12^. The steps of DNA methylation and demethylation are depicted in the diagram. While DNMT1 maintains these patterns during DNA replication, DNMT3A and DNMT3B allow de novo methylation, which adds methyl groups to cytosine. TET enzymes induce active demethylation, which transforms 5-methylcytosine into intermediates (such as 5-hydroxymethylcytosine), which TDG then processes to produce unmethylated cytosine. A breakdown in maintenance methylation results in passive demethylation, which causes a progressive loss of methylation. These systems guarantee epigenetic stability and dynamic control of gene expression (Fig. 2).

The most basic epigenetic change, DNA methylation is critical for controlling gene expression, maintaining genomic integrity, and maintaining chromatin structure. 5-methylcytosine (5-mC) is the result of this process, which is defined by the transfer of a methyl group to the cytosine’s fifth carbon within the context of cytosine-guanine (CpG)^13^. Primarily inside CpG dinucleotides, DNA methylation takes place in regions densely packed with these sequences, known as CpG islands (CGIs). These sequences are primarily found in promoter regions and are often unmethylated to preserve a permissive chromatin state for transcription^14^. A paradox of methylation patterns is seen in cancer: focused hypermethylation at gene promoter’s contrasts with widespread hypomethylation throughout the genome. Cancer is known for its genomic hypomethylation, which leads to instability. On the other hand, hypermethylation at tumor suppressor gene (TSG) promoters silences the gene by causing a shift to a more compact chromatin state^15^. The DNA methyltransferases (DNMTs), which use S-adenosylmethionine as a substrate, are the enzymatic architects of this methylome. DNMT1 and DNMT3A/3B, in particular, are primarily responsible for enforcing methylation patterns that preserve the status quo and establish new epigenetic signals. DNMT3A and DNMT3B are in charge of creating new methylation marks, whilst DNMT1 guarantees the faithful spread of methylation patterns throughout DNA replication. S-adenosylhomocysteine is also released as a result of this enzyme activity^16^. It is noteworthy that methylation is not limited to CpG dinucleotides; non-CpG methylation has been found in embryonic and brain contexts, highlighting the dynamic and intricate character of this epigenetic phenomena^15^. The ten-eleven translocation (TET) enzymes catalyze the conversion of 5-mC into a range of oxidized derivatives through a series of oxidation processes. This initiates a series of modifications that end with DNA demethylation. In this process, 5mC is gradually oxidized to 5-hydroxymethylcytosine (5hmC), followed by 5-formyl cytosine (5fC), and finally 5-carboxyl cytosine (5caC)^17^. As an alternative, the base excision repair machinery may delete 5fC and 5caC. These oxidized cytosines can be removed by DNA glycosylases, and subsequently, repair mechanisms restore an unmodified cytosine at these locations, therefore actively accomplishing demethylation^18^. DNA methylation is one epigenetic marker that is especially useful in disease studies since it persists over long periods and is a reliable predictor^19^. Interestingly, it has been demonstrated that the methylation state of individual CpG dinucleotides, as opposed to larger regions that contain numerous CpG dinucleotides, affects the regulation of gene expression and, consequently, the prognostic significance of a DNA methylation-based biomarker^20^.

Methylation of particular CpG dinucleotides within a single genomic region—like the promoter of a gene—can control transcription and determine if a biomarker has any clinical significance. It is crucial to determine a putative DNA methylation-based biomarker’s most pertinent chromosomal site. In addition to placement, the genomic context of DNA methylation needs to be considered. This includes things like the existence of distinct functional areas like enhancers or exons. Characteristic methylation profiles are linked to the existence of these distinct regions. For example, most gene bodies, especially exons, are heavily methylated, although promoter CpG islands are usually not^20^.

## Histone modification

**Fig. 3:**
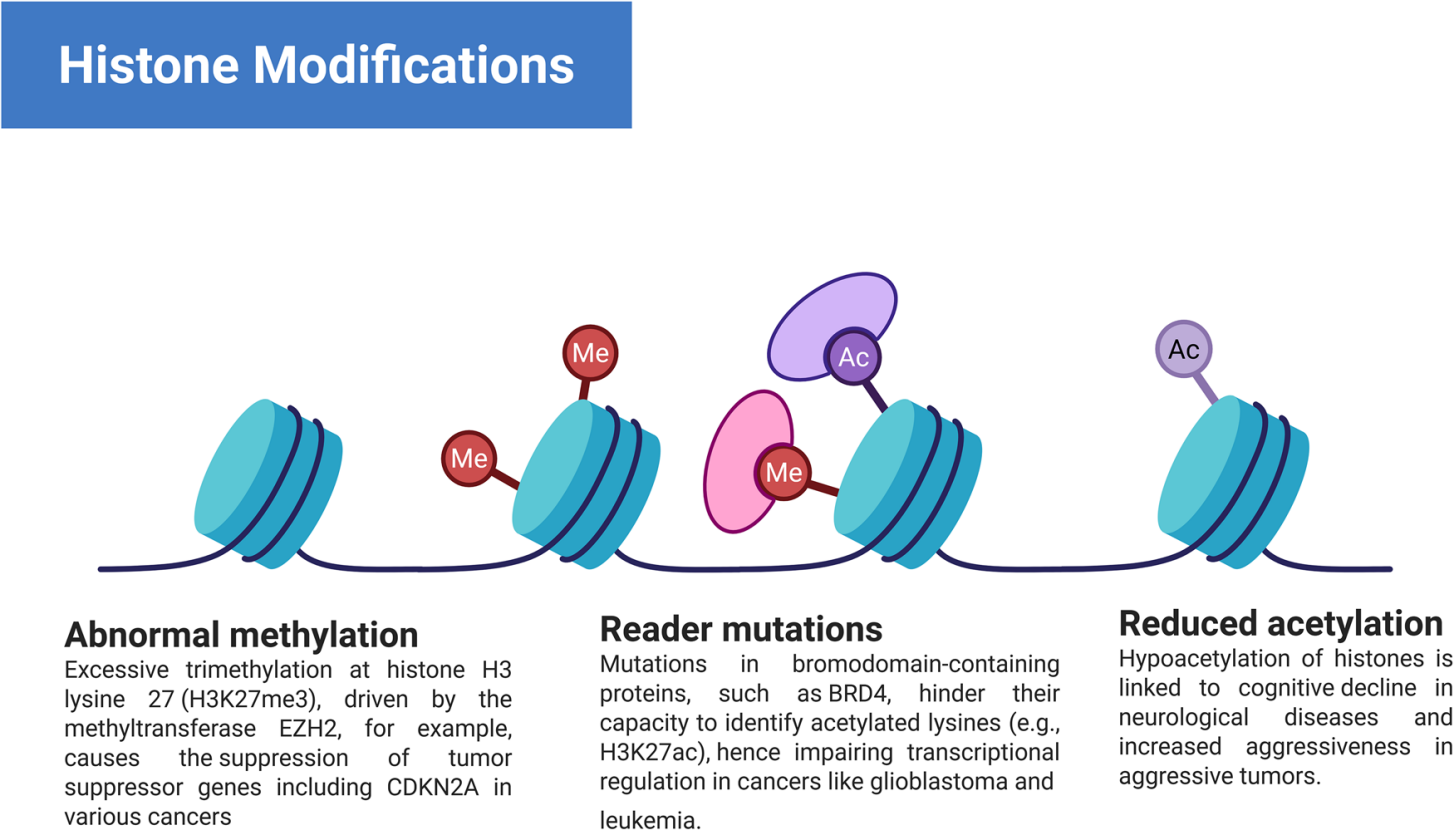
Histone modifications. Dysregulation of histone modifications in pathogenic conditions. This figure shows notable variations in histone post-translational modifications, which cause epigenetic dysregulation in several pathological states, including cancer. Atypical histone methylation is seen in the left panel. Excessive trimethylation at histone H3 lysine 27 (H3K27me3), driven by the methyltransferase EZH2, for example, causes the suppression of tumor suppressor genes including CDKN2A in various cancers. The main panel shows changes in histone modification “reader” domains that impair the ability to grasp certain histone marks. CBX family proteins’ chromodomain mutations, which are members of the Polycomb Repressive Complex 1, reduce H3K27me3 binding and cause abnormal gene expression. Mutations in bromodomain-containing proteins, such as BRD4, hinder their capacity to identify acetylated lysines (e.g., H3K27ac), hence impairing transcriptional regulation in cancers like glioblastoma and leukemia. The right panel shows lower histone acetylation, a sign of transcriptional suppression. A good example is the drop in H4K16ac, which corresponds with chromatin condensation and the downregulation of neuroprotective and DNA repair genes. Often seen in aged tissues, hypoacetylation of histones is linked to increased aggressiveness in aggressive tumors.

Key epigenetic changes that contribute to cancer include reduced acetylation, reader mutations, and abnormal DNA methylation patterns, such as hypermethylation of gene promoters, which can silence important genes involved in cancer cell growth and proliferation. Mutations in epigenetic readers, proteins that interpret methylation marks and other histone modifications, can disrupt the cellular response to these epigenetic signals, resulting in disrupted gene regulation and cancer progression. Certain cancers may also be exacerbated by decreased acetylation of histones and other proteins, which is a defining feature of epigenetic repression. This can impair chromatin accessibility and inhibit the expression of genes that maintain cancer cell function. The clinical characteristics of cancer, such as uncontrolled cellular growth, and metastasis, may be influenced by these coupled epigenetic alterations **(**Fig. 3**)**. Histones become essential components of the dynamic architecture of chromatin. The octameric core of these proteins is formed by the assembly of two copies of each of the histone variations H3, H4, H2A, and H2B. This group functions as a spool around which a complex 146-base-pair DNA sequence wraps^21^. The globular histones have tails that are rich in arginine and lysine, two basic amino acid bases that are prime for a wide range of covalent posttranslational modifications (PTMs). These chemical changes modify the way that histones bind with DNA, changing the chromatin landscape. They also produce docking sites for proteins that control the activity of chromatin^22^. Acetylation, methylation, and phosphorylation are the three main histone modifications that have been elucidated by a large body of research. However, a variety of other PTMs broaden the histone code and add to the intricate control of gene expression. These include but are not limited to lactylation, citrullination, ubiquitination, adenosine diphosphate (ADP)-ribosylation, and crotonylation.

### Histone acetylation

The equilibrium between histone deacetylases (HDACs) and histone acetyltransferases (HATs) controls the acetylation of histones. Acetylation can lower the lysine residues’ positive charge, which will prevent histone tails from adhering to negatively charged DNA and reveal the underlying DNA^23^. Histone acetylation is therefore typically regarded as an active histone mark^24^. Additionally, the same lysine residues’ acetylation and methylation can function as antagonists to block one another, causing crosstalk between various histone marks^25^. The key to this epigenetic regulation is the transfer of an acetyl group from the donor molecule acetyl-coenzyme A (acetyl-CoA) to these residues, highlighting the function of HATs in regulating chromatin shape and gene expression. Based on their primary structural homology, HATs can be classified into three major families: the MYST family, which includes TAT interacting protein 60 (Tip60) and monocytic leukemia zinc finger protein; the p300/CBP family, which includes p300 and CREB-binding protein (CBP); and the general control nonderepressible 5 (GCN5)-related N-acetyltransferase family, which is represented by GCN5 and p300/CBP-associated factor^26^. Four classes of human HDACs have been identified: class I Rpd3-like proteins (HDAC1-3 and HDAC8); class II Hda1-like proteins (HDAC4-7, HDAC9, and HDAC10); class III Sir2-like proteins (SIRT1-7); and class IV protein (HDAC11). Eleven HDACs are found in humans. A conserved set of amino acid residues at active sites unites class I and class II histone deacetylases (HDACs), which catalyze the metal-dependent hydrolysis of acetylated histones^27^. Class III HDACs require NAD+ to produce nicotinamide and the metabolite 2′-O-acetyl-ADP-ribose during the deacetylation process, whereas class I, II, and IV HDACs are zinc-dependent^28^. Numerous HDAC inhibitors have been created and studied for cancer. Animal tumor growth may be inhibited by them if they induce cell differentiation and/or death^29^.

Histone writers are the enzymes that produce histone marks. In metazoans, there are three main families of histone acetyltransferases (HATs): GNAT (GCN5-related N-acetyltransferase), MYST (Moz, Ybf2, Sas2, and Tip60), and p300/CREB-binding protein (p300/CBP). Histone crotonyltransferases (HCTs) include p300/CBP and the MYST family protein MOF; GNAT family protein GCN5 cannot catalyze histone crotonylation. A relatively new epigenetic marker is histone crotonylation, a post-translational modification defined by adding a crotonyl group to lysine residues on histone tails. It is functionally connected to active gene transcription and often points to the promoters and enhancers of highly activated genes. Unlike acetylation, crotonylation could have different influences on chromatin structure and gene control, affecting physiological processes including spermatogenesis, inflammation, and cancer advancement. Sabari et al. discovered that the coactivator p300 was both HAT and HCT, which was the first^30^. Additionally, they demonstrated that on recombinant chromatin templates, histone crotonylation directly triggered transcription more than histone acetylation. Many studies have been conducted on the mechanism of p300-mediated histone acetylation^31^.

Despite being a strong histone acetyltransferase, p300’s activity decreases with increasing acyl-CoA chain length and rigidity because of steric restraint. The histone acylation efficiencies for propionyl-CoA, butyryl-CoA, and crotonyl-CoA decrease to roughly 33%, 2.2%, and 1.5%, respectively, in comparison to acetyl-CoA^32,33^.

There are four categories for histone deacetylases (HDAC). Class III HDACs, also known as sirtuins, are NAD-dependent, whereas class I, class II, and class IV HDACs are Zn-dependent. It has been observed that histone decrotonylase (HDCR) and histone de-β-hydroxybutyrylase activities are present in both class I and class III HDACs. The first evidence of HDCR activity in vitro was found for HDAC3^34^. Madsen and Olsen discovered that HDAC3-NCoR1 could catalyze crotonylated substrates in vitro using a variety of fluorogenic substrates, however, the effectiveness was significantly lower than that of acetyl groups^34^. Class I HDACs (HDAC1, HDAC2, HDAC3, and HDAC8) were recently found to be the primary HDCRs in cells and to have distinct site-specificity from SIRT1^35^. To date, no class I HDAC complex structure using a crotonylated histone substrate has been determined.

### Histone methylation

PTM, known as histone methylation only affects arginine, lysine, and histidine residues. It does not affect the charge of proteins. Arginine can obtain one or two methyl groups, either symmetrical or asymmetrical, whereas lysine can gain up to three methyl groups^36^. Because lysine methylation on histones has a distinct regulatory role that is regulated by specific effector molecules recognizing methyl marks rather than by changing histone charge, it has attracted a great deal of study interest^37^. Even though histone methylation was first identified in the 1960s, the importance of histone lysine methylation in regulatory roles was only recently highlighted^38^. Cancer progression can result from an imbalance in the processes of histone methylation and demethylation, highlighting the importance of these epigenetic regulators^39^. Based on their precise position and methylation status, histone lysine methylations carry complex codes that affect transcriptional outcomes. While certain methylations indicate transcriptional activity, others are suggestive of gene silence. For example, active transcription is typically linked to methylations at H3K4, H3K36, and H3K79, while restrictive chromatin states are connected with methylations at H3K9, H3K27, and H4K20^40^. These modifications are known to interact with DNA methylation and other histone modifications to fine-tune gene expression; they do not function in isolation. For instance, in yeast, methylations at H3K4 and H3K79 depend on H2B’s previous ubiquitylation, exposing a network of interrelated epigenetic regulation^41^.

## Chromatin remodeling

The ATP-hydrolyzing chromatin remodeling complexes (CRCs) are essential for determining DNA packing. These complexes manipulate the accessibility of the genetic code by sliding nucleosomes, changing histone variations, and adding or removing histones. This allows them to attain regulatory finesse^42^. Based on similarities in their catalytic ATPase cores and related components, mammalian CRCs can be divided into four primary classes: the switch/sucrose non-fermentable (SWI/SNF), imitation SWI (ISWI), chromodomain helicase DNA-binding (CHD), and inositol requiring 80 (INO80) families^43^. The SWI/SNF family, which has eight to fourteen subunits and was first identified through research on Saccharomyces cerevisiae, is essential for changing chromatin from a quiescent to an active state. It accomplishes this by permitting nucleosome relocation and controlling the insertion and removal of histone octamers. The SWI/SNF family splits into three subfamilies in mammals: the non-canonical BAF, polybromo-associated BAF (PBAF), and canonical BAF (cBAF)^44^. Even though SMARCC1, SMARCC2, SMARCD1, and the ATPases SMARCA4 or SMARCA2 are shared core subunits, each complex has its constellation of extra subunits that give them their own functional identities^45^. Prototypical ISWI complexes that improve nucleosome organization and nucleosome spacing, such as ATP-utilizing chromatin assembly and remodeling factor (ACF) and chromatin accessibility complex (CHRAC), are known to promote transcriptional repression^44^. RNA polymerase II activation and transcriptional activation are facilitated by the disruption of nucleosome spacing caused by entities like the NURF complex, which defies this tendency^46^. Some CHD remodelers, like the Mi-2/nucleosome remodeling and deacetylase (NuRD) complex, are linked to transcriptional inhibition, while others, like some known to increase transcription by sliding nucleosomes or deconstructing them completely^47^. While this is going on, the INO80 complex is essential for transcriptional upregulation and DNA repair^48^.

Recent studies have shed light on the complex regulatory environment controlling the abnormal behavior of cancerous stem cells. The discovery of bi-allelic inactivating mutations in the gene encoding the hSNF5 subunit of the SWI/SNF complex, a characteristic of malignant rhabdoid tumors, is at the center of this discussion^49^. The functional dynamics of ATP-dependent chromatin remodelers are influenced by extracellular and intracellular stimuli in addition to genetic modifications. Notably, in response to the cellular DNA damage signaling, there is an increase in the ATPase-driven nucleosome repositioning activity^50^. Findings indicating miR-221 downregulates the SWI/SNF component ARID1A^51^ provided more evidence for post-transcriptional control. However, this effect is mitigated by the long non-coding RNA (lncRNA) CASC15, which sequesters miR-221 and lessens its suppressive effect^52^. Furthermore, post-translational modifications play a role in the regulation of CRCs in cancer, as demonstrated by the ubiquitination-induced degradation of SMARCA4—a process that is mediated by the E3 ubiquitin ligase complex and has implications for the inhibition of gastric cancer metastasis^53^. Taken together, these findings outline a multifaceted network of regulatory inputs that customize CRC activity throughout cancer progression.

DNA is carefully arranged around histone octamers inside the nucleus to produce nucleosomes, which are the fundamental building blocks of chromatin architecture^54^. Initial structuring gives rise to a wide range of three-dimensional chromatin configurations, such as loops, topologically associating domains (TADs), lamina-associated domains (LADs), and blocks (A/B compartments). These configurations are coherently mediated by structural proteins, including RAD21, CTCF, and structural maintenance of chromosomes (SMC)^55^. These complex structures are not just structural; they also have a significant impact on the control of the cell cycle, DNA replication, and developmental processes. They also play a crucial role in modifying gene expression and cellular identity^56^. Increased intra-domain interactions set TADs apart as independent chromosomal areas^57^. TAD demarcation is led by CTCF, a nuclear phosphoprotein that has been evolutionarily conserved and is prominently located at TAD boundaries^58^. CTCF directly interacts with cohesin to orchestrate the formation and maintenance of TADs and chromatin loops.

LADs are sections of the genome that are closely linked to the nuclear lamina in the architecture of the cell nucleus. The nuclear lamina is a structural network made primarily of laminas, or V-type intermediate filament proteins, that stick to the inner nuclear membrane^59^. These domains have an intrinsically repressive regulatory environment: genes in LADs are usually low expressed, and this is correlated with enrichment of di- and tri-methylated histone H3 lysine 9 (H3K9me2 and H3K9me3) marks, which are indicators of transcriptional silence^60^.

## The Role of ncRNAs in Epigenetic Regulation and Oncogenesis

Non-coding RNAs (ncRNAs), especially microRNAs (miRNAs) and long non-coding RNAs (lncRNAs), are being more recognized as important controllers of epigenetic mechanisms. These substances influence gene expression at many levels, including chromatin remodeling, transcriptional control, and post-translational modifications. In the cancer field, ncRNAs often significantly impact oncogene activity and the silence of tumor suppressor genes.

Generally, 20–24 nucleotides long, microRNAs (miRNAs) operate by attaching to matching sequences on target messenger RNAs (mRNAs), causing mRNA destruction or translational suppression. In glioblastoma (GBM), dysregulated miRNAs show oncogenic or tumor-suppressive effects. Prominent oncomiR miR-21, for instance, is overexpressed in glioblastoma multiforme (GBM) and colorectal cancer (CRC), where it suppresses the expression of tumor suppressors like PTEN and PDCD4, therefore promoting greater proliferation, invasion, and chemoresistance^61,62^. By contrast, GBM generally downregulates tumor-suppressive miRNAs, such as miR-34a, promoting stemness and vulnerability to apoptosis^63^.

Exceeding 200 nucleotides in length, long non-coding RNAs (lncRNAs) control epigenetic states via interacting with chromatin modifiers. A good example is the lncRNA HOTAIR, which draws Polycomb Repressive Complex 2 (PRC2) to specific genomic locations, causing gene suppression by trimethylation of histone H3 at lysine 27 (H3K27me3). HOTAIR is significantly higher in both GBM and CRC, with poor prognosis and increased aggressiveness^64,65^. The lncRNA MALAT1 significantly contributes to metastatic development in colorectal cancer. It promotes epithelial-to-mesenchymal transition (EMT) and helps to enable alternative splicing^66^.

Both ncRNAs indirectly influence epigenetic states by altering the expression of epigenetic enzymes, including DNMTs, HDACs, and EZH2. Members of the miR-29 family in GBM are known for their capacity to downregulate DNA methyltransferases (DNMT3A and DNMT3B), hence causing DNA hypomethylation and the reactivation of silenced tumor suppressor genes^67^. An increasing area of research with consequences for therapy resistance and tumor plasticity is the interaction between lncRNAs and histone-modifying enzymes.

## Histone variant H3.3 and its mutations in pediatric gliomas

Produced by the H3F3A and H3F3B genes, the histone variant H3.3 is essential for controlling chromatin structure and gene expression during development and cellular differentiation. Unlike traditional histones, H3.3 is incorporated into chromatin regardless of DNA replication, often indicating actively transcribed regions^68^. In pediatric high-grade gliomas (pHGG), including diffuse intrinsic pontine gliomas (DIPG) and glioblastomas, somatic mutations in H3.3, particularly at lysine 27 (K27M) and glycine 34 (G34R/V), have been acknowledged as major oncogenic drivers.

The H3.3K27M mutation changes the usual post-translational modification environment by replacing lysine with methionine at position 27. Dominant inhibition of EZH2, the methyltransferase component of PRC2, induces a global drop in the repressive H3K27me3 mark, hence reprogramming the epigenome and enabling carcinogenic transcriptional pathways^69,70^. Despite the global decline in H3K27me3, specific gene promoters might ironically maintain or gain H3K27me3 marks, hence creating a complex restrictive environment at specific loci^71^.

Apart from K27M, G34 (e.g., G34R/V) mutations affect relationships with chromatin-binding proteins and correspond with particular tumor locations and age groups. G34 mutations impede the histone methyltransferase SETD2’s recruitment, reducing H3K36 trimethylation (H3K36me3), an alteration linked to active transcription and genomic stability^72^. These changes have been linked to the development of genomic instability and the alteration of differentiation pathways in brain progenitor cells.

These histone changes indicate potential therapy targets and act as diagnostic markers. For example, studies on EZH2 inhibitors or drugs changing PRC2 activity aim to fix the epigenetic dysregulation brought on by K27M mutations^73^. Furthermore, recent studies show that H3.3 mutations could change the character of the immunological environment, opening possibilities for immunotherapeutic approaches^74^.

## Epigenetics changes in cancer

**Fig. 4:**
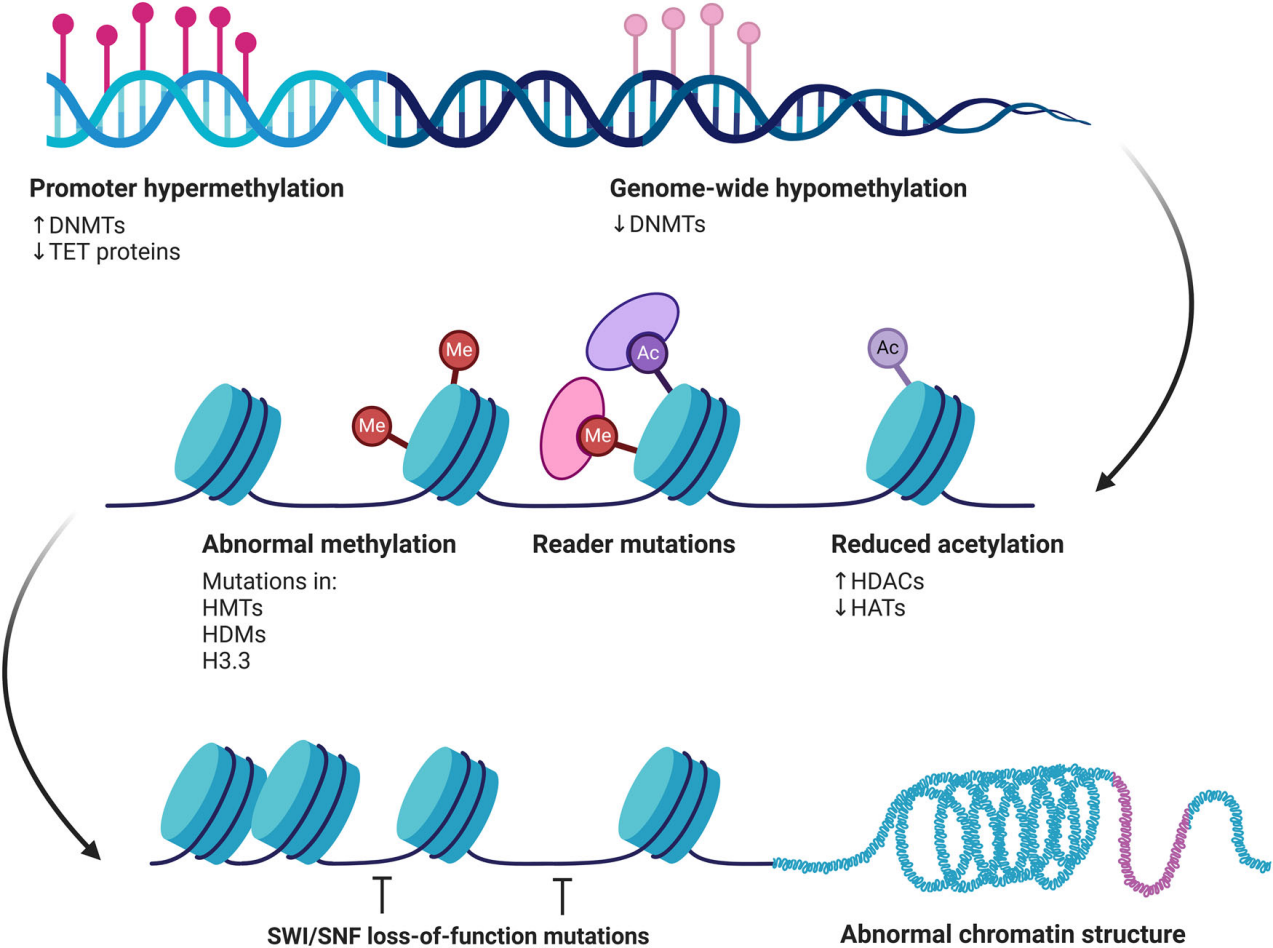
Cancer epigenetics. DNA methylation, histone modifications, and chromatin remodeling are the three main epigenetic changes that drive the progression of cancer. DNA methylation changes include promoter hypermethylation, which suppresses tumor suppressor genes because of increased DNMT activity and decreased TET protein activity; on the other hand, genome-wide hypomethylation, which is frequently caused by decreased DNMT activity, causes genomic instability and oncogene activation; histone modifications include mutations in Histone Methyltransferases (HMTs), Histone Demethylases (HDMs), and histone variants like H3.3, which result in abnormal methylation patterns; and reduced histone acetylation, which further suppresses normal gene expression and contributes to cancer. Defects in chromatin remodeling, including loss-of-function mutations in SWI/SNF complexes, produce condensed chromatin structures that impair regular gene regulation and increase the likelihood of tumor development. These linked alterations result in aberrant chromatin architecture and dysregulated gene expression, which are characteristics of cancer^194^.

The three primary epigenetic alterations that propel cancer development are DNA methylation, histone modifications, and chromatin remodeling. Histone modifications include mutations in HMTs, HDMs, and histone variants like H3.3, which result in abnormal methylation patterns; reduced histone acetylation, which further suppresses regular gene expression and contributes to cancer; and promoter hypermethylation, which suppresses tumor suppressor genes due to increased DNMT activity and decreased TET protein activity. Conversely, genome-wide hypomethylation, which is often caused by decreased DNMT activity, causes genomic instability and oncogene activation. Condensed chromatin structures caused by chromatin remodeling defects, such as loss-of-function mutations in SWI/SNF complexes, hinder regular gene regulation and raise the risk of tumor growth. These interconnected changes lead to dysregulated gene expression and abnormal chromatin architecture, both of which are hallmarks of cancer (Fig. 4).

Normal growth, development, and organ-specific gene expression depend on epigenetic mechanisms. Nonetheless, abnormal changes to the epigenome are essential to the etiology of disease, including cancer. Different chemical alterations, such as differences in the expression of genes and histone-modifying enzymes, vary throughout cell types and are closely linked to different forms of cancer. These chemical changes lead to changed patterns of gene expression, which impact cellular properties including proliferation and invasiveness^75^. Important alterations include aberrant DNA methylation that suppresses TSGs and activates oncogenes^76^; histone modifications that affect gene expression and chromatin structure; chromatin remodeling that affects how essential genes are transcriptionally regulated; and changes to higher-order chromatin structure that change the spatial relationships between genes and regulators^77^.

Widespread hypomethylation of DNA is associated with chromosomal instability and oncogene activation, while hypermethylation of CGIs is associated with TSG repression due to chromatin remodeling towards a repressive state. These hypomethylated domains exhibit notable overlap with regions generally characterized by restrictive chromatin that are dramatically reduced in cancer cells, such as large organized chromatin K-modifications (LOCKs) and large organized domains (LADs)^78^. An important turning point was the identification of DNA methylation in the retinoblastoma TSG (RB1) promoter region. Since then, many TSGs have been discovered, such as DNA repair proteins like MutL Homolog 1 (MLH1), cell cycle inhibitors like cyclin-dependent kinase inhibitor 2 (CDKN2), angiogenesis blockers like Von Hippel-Lindau (VHL) tumor suppressor, and cell adhesion molecules like CDH1 (cadherin-1)^79^. Notably, many genes exhibit tissue-specific DNA hypermethylation that is reminiscent of germline abnormalities found in malignancies that run in families^80^. It is believed that methyl-CpG-binding (MBD) proteins attract transcriptional repressors or histone-modifying enzymes as part of the mechanism of promoter hypermethylation-induced gene silence. The discovery of the NuRD complex, which contains MBD2 and has been demonstrated to bind to and mute genes like p14/p16 in cancer cells, has given support to this theory^81^.

Through their modulation of transcriptional activity, histone alterations have a role in the initiation and growth of tumors, frequently leading to the overexpression of oncogenes and the downregulation of TSGs. H3K27me3 alterations are a prime example of this dysregulation, as they have a significant effect on genomic stability^82^. Differences in the H3K27me3 status are caused by several dysfunctions, such as recurrent mutations that either improve or worsen the function of the gene that codes for the methyltransferase that catalyzes this particular histone modification, the enhancer of zeste homolog 2 (EZH2) gene^83^.

Heterozygous somatic mutations have been shown to produce binding sites for the MYB transcription factor upstream of the TAL1 oncogene in acute lymphoblastic leukemia (ALL). Because of the attraction of CBP by this MYB interaction, a super-enhancer is formed, which in turn promotes the overexpression of TAL1 and leukemogenic transformation^84^. The Sirtuin family of NAD + -dependent deacetylases has also been shown to be important regulators of epigenetic alterations such as desuccinylation, demalonylation, and deacetylation. This includes mitochondrial SIRT3, SIRT4, and SIRT5^85^. Loss of SIRT4 has been shown to increase the capacity of breast cancer stem cells for self-renewal. SIRT4 is essential for the catabolism of nutrients^86^. Increased cancer cell proliferation has been connected with desuccinylation and a consequent decrease in succinate dehydrogenase (SDH) activity, which is correlated with high levels of SIRT5 activity. On the other hand, SDH hyper-succinylation and reactivation result from suppressing SIRT5, which suppresses the proliferation of cancer cells^87^.

Since mutations in the genes of the SWI/SNF complex are detected in more than 20% of malignancies, indicating their significance in carcinogenesis, CRCs, in particular the SWI/SNF family, have been thoroughly examined and are essential to the DNA damage response (DDR)^88,89^. The SWI/SNF complexes affect DNA repair pathways by increasing nucleosome mobility via ATPase activity, which promotes DDR. SWI/SNF subunits have been found to play a variety of roles in DDR; some alter the chromatin architecture at DNA damage sites, while others directly attract DDR proteins. Non-homologous end joining and homologous recombination are two DNA repair processes that have been linked to the cBAF and PBAF complexes^4^. To be more precise, SMARCA4 and the cBAF-only ARID1A are drawn to DNA lesions to help in double-strand break resolution and repair^90^. Additionally, it has been demonstrated that SMARCA4 collaborates with poly-ADP ribose polymerase 1 (PARP1) at damage sites, promoting chromatin remodeling to lower nucleosome density and assisting in the healing process^91^. It is possible that SMARCA4 and ARID1A also play a role in DNA decatenation and telomere cohesion because their deficiencies have been linked to aberrant chromosomal segregation and mitotic abnormalities^92^. PBRM1, a PBAF component, is also linked to DDR; it is hypothesized that it plays roles in centromeric cohesion maintenance, which is essential for preserving genomic integrity, and transcriptional suppression at double-strand breaks to speed DNA lesion repair^93^.

The process of metastasis requires that cancer cells move from their original site, travel through the bloodstream, become resistant to hemodynamic forces, adapt to the unique cellular microenvironment at the secondary site, and avoid strong immune cell interactions, all of which have significant impact on the cancer cell’s ability to survive^94^. Cancer cells must undergo the epithelial-mesenchymal transition (EMT) to acquire mesenchymal properties. During this process, the cells must give up their epithelial characteristics^7^. Notably, enhanced invasiveness of thyroid cancer cells in vitro and the suppression of E-cadherin in lymph node metastases of papillary thyroid carcinoma are linked to the epigenetic downregulation of CDH1. Elevated TGF-β–Smad2 pathway activation and DNA methylation-mediated CDH1 silencing are consistent in breast cancer, suggesting a complicated interaction between signaling pathways and epigenetic alterations in the narrative of cancer metastasis^95^.

Histone acetylation patterns on H3 and H4 have emerged as distinctive markers of cancer cells, and dysregulation in the landscape of histone modifications is becoming more and more linked to metastasis. Histone acetylation states in cancer are impacted by metabolic reprogramming, which modifies absolute acetyl-CoA and the ratio of acetyl-CoA to coenzyme A. Owing to its ability to produce acetyl-CoA via ligating acetate and CoA, ACSS2 can cause HIF-2α to become acetylated, which inhibits EMT in Hepatocellular Carcinoma (HCC) when oxygen levels are low^96^. The primary enzyme in the liver that selectively hydrolyzes the thioester bond of acetyl-CoA to produce acetate and CoA is called cytoplasmic acetyl-CoA hydrolase, or acetyl-CoA thioesterase 12 (ACOT12)^97^. Reduced ACOT12 levels in HCC cause acetyl-CoA levels to rise, which in turn promotes acetylation of H3K9 and H3K56, facilitating TWIST2-mediated EMT^98^.

Recent comprehensive functional and mechanistic investigations have provided new insights into the intricate process of tumor metastasis, identifying disruptions in chromatin remodeling as major drivers^99^. SMARCA4, a chromatin remodeler, has been confirmed to function as a tumor suppressor. Its reduced expression has been associated with increased colorectal cancer metastasis through the Wnt/β-catenin signaling pathway^100^. Furthermore, it has been demonstrated that ARID1A attenuation promotes liver cancer cell metastasis via interfering with the SMARCA4–RAD21 interaction^101^.

The SWI/SNF core component BAF155 promotes tumor growth and metastasis in breast cancer. By targeting key genes essential for metastasis, arginine methyltransferase 4 (PRMT4) methylates BAF155 and repositions it throughout the genome^102^. Recent research has shed light on the critical function that histone variation incorporation into chromatin plays during metastatic colonization, adding another level of complexity. In particular, the histone chaperone complex CAF-1 integrates the histone H3 variation H3.3 into chromatin, facilitating chromatin accessibility and initiating a transcriptional program that promotes aggressive tumor behaviors and metastatic growth^103^. These results highlight the critical roles that histone chaperones and chromatin remodelers play in determining the fate of cancer cells, and they offer these proteins as potential targets for therapeutic intervention in the treatment of invasive malignancies.

In cancer, epigenetic and metabolic changes are tightly entwined and mutually regulated. Metabolites can alter the epigenetic landscape by acting as cofactors or substrates for enzyme processes. For instance, under physiological settings, the concentration of acetyl-CoA can dynamically regulate the process of histone acetylation. In cancer, metabolic reprogramming modifies the ratio of acetyl-CoA to coenzyme A and absolute acetyl-CoA, which in turn impacts the acetylation statuses of histones. When caspase-10 cleaves ACLY, it lowers intracellular lipid levels and inhibits the acetylation of H3 and H4 by GCN5, which in turn prevents the expression of genes linked to tumor growth and metastasis^104^. AKT, mTOR, and phosphoinositide 3-kinase (PI3K) are examples of metabolic signaling pathways where DNA methylation also leads to the silence of important TSGs. These pathways are essential for the activation of glycolysis and the specialized metabolism of cancer cells. In cancer, tumor suppressors that block the function of these signaling pathways, like liver kinase B1, phosphatase and tensin homolog (PTEN), and VHL, are frequently epigenetically silenced, which causes their metabolic reprogramming^105–107^.

The pivotal function of the SWI/SNF complex in the metabolic reprogramming attribute of oncogenesis has been elucidated by a recent study. In particular, it has been determined that the ARID1A component directly regulates gene glutaminase 1 (GLS1), which is essential for the metabolism of cancer. When the ARID1A function is lost, the GLS1 promoter becomes more accessible, which increases the production of glutaminase. Because of this alteration, clear cell ovarian cancer cells become more dependent on glutamine metabolism for the synthesis of aspartate and nucleotides as well as for reduced glucose uptake^108^. Similar discoveries in lung cancer demonstrate that mutations in SMARCA4, an additional SWI/SNF component, influence gene regulation in response to glycolysis and hypoxic stress, presumably as an adaptive response to energy strain. Due to increased fatty acid and protein synthesis, these changes in SMARCA4-mutant cells cause a heightened need for energy. This differs from the typical Warburg effect in that the tumor’s energy metabolism switches from glycolysis to oxidative phosphorylation^109^. The metabolic rearrangement in IDH-mutant gliomas and changed CpG methylation patterns have been linked in recent research^110^. DNA hypermethylation at CpG islands is noticeably common in these tumors. The CTCF-binding sites that are closely linked to these differentially methylated regions (DMRs) are essential to this epigenetic rearrangement. Given that CTCF is well-known for its function in anchoring genomic loops, it is noteworthy that a sizable fraction of DMRs found coincides with chromatin loop anchorage sites. The ability of SDH mutations to produce de novo DMRs within the genome is matched by 77,213 IDH mutations. This is achieved by the accumulation of metabolites, particularly α-ketoglutarate and the oncometabolite 2-hydroxyglutarate, which obstruct the activity of histone and DNA demethylases belonging to the TET family^111^.

Recent developments emphasize how crucial metabolic processes directly affect the activity of chromatin-modifying enzymes by regulating the availability of their cofactors or substrates. Several intermediates directly influence DNA and histone methylation; hence the tricarboxylic acid (TCA) cycle is crucial to this epigenetic-metabolic connection. An important metabolite, alpha-ketoglutarate (alpha-KG), is a cofactor for the TET family of DNA demethylases and Jumonji C (JmjC) domain-containing histone demethylases. Disruption of TCA cycle enzymes, such as succinate dehydrogenase (SDH) or fumarate hydratase (FH), causes the accumulation of succinate and fumarate, which act as competitive inhibitors of α-KG-dependent dioxygenases. These metabolic changes create a repressive chromatin environment that silences tumor suppressor genes and drives oncogenesis by causing genome-wide hypermethylation of DNA and histones^112–114^.

At the same time, glycolysis is essential for histone acetylation modulation via acetyl-CoA, the key donor molecule for histone acetyltransferases (HATs). The conversion of glucose to pyruvate in glycolytic tumor cells controls acetyl-CoA levels via pyruvate dehydrogenase (PDH) and ATP-citrate lyase (ACLY), among other enzymes, so that it can enter mitochondria or be converted to lactate. Active glycolysis increases cytosolic acetyl-CoA levels using increased ACLY activity, hence enhancing histone H3 and H4 acetylation and driving the transcription of pro-proliferative and anti-apoptotic genes^115,116^. Moreover, hypoxia—often seen in solid tumors—aids the PDK-mediated suppression of PDH, hence changing acetyl-CoA metabolism and chromatin acetylation in favor of transcriptional programs supporting malignancies^117,118^.

Glutaminolysis, which supports energy generation and the creation of substrates like alpha-KG and nucleotide precursors, is another important activity. Often, tumors with high glutamine need to affect epigenetic programming by altering alpha-KG levels. In MYC-driven cancers, more glutamine flow helps α-KG-dependent histone demethylation at oncogenic sites, hence encouraging tumor development^119,120^ On the other hand, in situations of nutrient deprivation or therapeutic resistance, a drop in α-KG level can block demethylase activity, causing the accumulation of repressive histone modifications like H3K9me3 and H3K27me3, which supports a more stem-like or quiescent state favorable for tumor survival^121,122^.

By generating S-adenosylmethionine (SAM), the universal methyl group donor, the methionine cycle and one-carbon metabolism greatly influence DNA and histone methylation. Cancer cells showing increased flux through the methionine and folate cycles—often driven by upregulated enzymes such as MAT2A and MTHFD1L—exhibit higher SAM levels, therefore enabling global DNA hypermethylation and selective repression of differentiation-related genes^123,124^. Inhibition of SAM synthetase or methionine restriction has been shown to lower histone H3K4 and H3K9 methylation, hence impeding cancer cell growth and promoting differentiation^125–127^.

Eventually, data shows that lipid metabolism is a key regulator of epigenetic states. Especially in metabolic circumstances defined by limited glucose supply, fatty acid oxidation (FAO) provides acetyl-CoA for histone acetylation. Histone acetylation is reduced by inhibiting fatty acid oxidation enzymes—especially CPT1A—and the expression of genes linked to oxidative metabolism and cellular survival is therefore hampered 127. Furthermore, specific lipid-derived metabolites, such as \u03b2-hydroxybutyrate, act as natural HDACs inhibitors, therefore promoting histone hyperacetylation and increasing the transcription of anti-inflammatory or neuroprotective genes under particular circumstances, including caloric restriction or ketogenic diets^128,129^.

An ecosystem that is both dynamic and complicated, the Tumor Microenvironment (TME) is essential to the growth of tumors. It includes a variety of non-cancerous cellular elements within a vascularized extracellular matrix in addition to the cancer cells. Numerous immune cells, cancer-associated fibroblasts (CAFs), endothelial cells, pericytes, and tissue-specific cells like adipocytes and neurons are all part of this network^130^. Changes in the DNA methylation landscape serve as an example of the substantial epigenetic remodeling that occurs in cancer cells and the stromal inside the TME. This shows up as both the above-described targeted hypermethylation of TSGs and extensive hypomethylation of the genome. The primary matrix component of the TME, CAFs, appears to have distinct methylation patterns that are indicative of the cancer type that it is linked to^131^. For example, prostate cancer CAFs show both hypo- and hypermethylation at certain gene loci, whereas CAFs from stomach, colon, and lung malignancies are characterized by global hypomethylation and gene-specific hypermethylation^5,132^. In many types of cancer, aberrant methylation is frequently accompanied by DNMT1 overexpression, which is fueled by signaling cascades controlled by cancer cells^133,134^. Not only do cancer cells exhibit abnormal expression of histone-modifying enzymes, but the stromal cadre of the TME also does. A spike in particular HDACs influences a number of immunological and structural changes in CAFs. The recruitment of MDSCs and Treg cells is facilitated by increased prostaglandin E2 (PGE2)/cyclooxygenase-2 (COX2) signaling, which is catalyzed by overexpression of HDAC6. In a similar vein, higher HDAC1, HDAC3, and HDAC8 levels in CAFs are associated with enhanced ECM secretion, which affects immune surveillance^135^. The metastasis-associated protein 1 (MTA1), a crucial component of the NuRD complex, has been found to be often overexpressed in a variety of malignancies, providing additional insight into the function of chromatin remodeling. Tumor progression is associated with overexpression of MTA1, which is characterized by a decrease in macrophages and a change in the immunosuppressive phenotype of the remaining macrophages, as well as a suppression of cytotoxic T lymphocyte activation, which results in an immune-tolerant TME. It is clear that more research is necessary given the significant effects of these CRCs on the immunological environment of the TME and the ensuing effects on immunotherapeutic resistance^136^.

## Epigenetic dysregulation in breast cancer and acute myeloid leukemia: disease-specific insights

Anchoring the subject of epigenetic dysregulation in particular cancer types, such as breast cancer and acute myeloid leukemia (AML), provides mechanistic clarity and therapeutic relevance. These cancers show how particular epigenetic changes such as aberrant DNA methylation and histone modification anomalies drive cancer development, progression, and treatment resistance.

Significant research has made clear in breast cancer the influence of abnormal histone alterations on the epigenetic environment of cancer cells. Aberrant histone acetylation and methylation patterns often reduce tumor suppressor genes and activate oncogenes. Overexpression of histone deacetylases (HDACs), particularly HDAC1 and HDAC6, for instance, has been linked to the promotion of aggressive phenotypes by means of chromatin condensation and transcriptional suppression of genes connected to cell cycle arrest and apoptosis. Similarly, the methyltransferase EZH2, the enzymatic component of PRC2, is often upregulated in triple-negative breast cancer (TNBC) and promotes oncogenesis by catalyzing the trimethylation of histone H3 at lysine 27 (H3K27me3), so suppressing tumor suppressor genes like CDKN1C^137,138^. In basal-like subtypes, mutations or functional deletion in the histone demethylases KDM6A/B, which reduce EZH2-mediated repression, exacerbate this imbalance and correspond with poor prognosis^139^.

Moreover, histone changes interact with breast cancer hormone receptor signaling. Though the lack of this control causes resistance to endocrine treatment, the estrogen receptor (ER) increases the transcription of estrogen-responsive genes by binding histone acetyltransferases (HATs) such p300/CBP. The relationship between histone modifiers and estrogen receptor status acts as both a biomarker for disease progression and a targetable vulnerability. Broad and recurrent changes in DNA methylation define epigenetic dysregulation in acute myeloid leukemia (AML). Roughly 20–30% of AML patients have DNA methyltransferase 3 A (DNMT3A) mutations linked with poor prognosis and chemoresistance^140^. Often, these mutations cause global hypomethylation and localized hypermethylation at tumor suppressor gene promoters, which disturb the hematopoietic program and compromise differentiation. Common in both acute myeloid leukemia (AML) and myelodysplastic syndromes, TET2 mutations drive hypermethylation patterns similar to those produced by DNMT3A loss-of-function mutations using DNA demethylation by conversion of 5-methylcytosine to 5-hydroxymethylcytosine (Figueroa et al. 2010).

One significant epigenetic feature of AML is the presence of fusion proteins, indicated by MLL (KMT2A) fusion proteins. These proteins change histone methylation patterns by recruiting DOT1L, a methyltransferase responsible for H3K79 methylation. This causes the ongoing activation of leukemogenic transcriptional pathways, including HOXA gene clusters^141^. Pharmacological inhibition of DOT1L has shown promise in preclinical and early clinical studies, therefore stressing the therapeutic potential of addressing epigenetic dependencies in AML^142,143^.

These epigenetic changes not only promote disease progression but also increase therapy resistance. Hypomethylating medicines, including azacitidine and decitabine, work as main therapies for some subtypes of AML145 by reversing promoter hypermethylation, reactivating repressed tumor suppressor genes, and promoting differentiation. Often, resistance develops through compensatory epigenetic reprogramming or clonal evolution, which calls for combinatorial strategies using HDAC inhibitors, BET bromodomain inhibitors, or immunomodulatory medications. A list of key epigenetic alterations across selected cancer types is provided in Table 1.

**Table 1:** Key Epigenetic Alterations Across Selected Cancer Types

Cancer Type	Primary Epigenetic Alteration	Molecular Target / Modifier	Effect / Outcome	References
**Breast Cancer**	Histone methylation ( ↑ H3K27me3)	EZH2 (PRC2 complex)	Silencing of tumor suppressor genes (e.g., *CDKN1C*)	Kouzarides, 2007^137^; Chang & Hung, 2012^138^
	Histone deacetylation	HDAC1, HDAC6	Chromatin condensation, repression of apoptotic/cell-cycle genes	Lin et al.^195^
	Histone acetylation imbalance	p300/CBP (HATs)	Estrogen receptor (ER) co-activation; therapy resistance	Skrzypczak et al.^196^
**Acute Myeloid Leukemia (AML)**	DNA methylation ( ↑ promoter hypermethylation)	DNMT3A (mutation)	Silencing of differentiation genes; poor prognosis	Ley et al.^140^
	DNA demethylation defect	TET2 (mutation)	Global DNA hypermethylation; blocked differentiation	Figueroa et al.^197^
	Histone methylation ( ↑ H3K79me)	DOT1L (recruited by MLL fusion)	Activation of leukemogenic HOXA genes	Deshpande et al.^198^; Daigle et al.^142^
**Glioblastoma**	DNA hypomethylation & enhancer reprogramming	IDH1 mutation	CpG island hypermethylator phenotype (G-CIMP); transcriptional reprogramming	Turcan et al.^199^
	Histone mutation (H3.3 K27M)	H3F3A (mutation)	Impaired PRC2 binding; global H3K27me3 loss; chromatin de-repression	Lewis et al.^69^
**Colorectal Cancer**	Promoter hypermethylation	MLH1	Mismatch repair deficiency; microsatellite instability (MSI)	Toyota et al.^200^
	miRNA-mediated gene silencing	miR-21, miR-135b	Inhibition of PTEN, APC; promotion of Wnt and PI3K signaling	Schetter et al.^201^
**Prostate Cancer**	Histone demethylation defect	LSD1 (KDM1A)	Activation of AR signaling; promotes tumor growth	Metzger et al.^202^
	DNA hypermethylation	GSTP1 (promoter)	Loss of detoxifying enzyme; early cancer biomarker	Yegnasubramanian et al.^203^
**Pediatric Gliomas**	Histone variant mutation (H3.3 G34R/V, K27M)	H3F3A	Altered chromatin remodeling; developmental gene dysregulation	Wu et al.^204^; Lewis et al.^69^

## Clinical trials and epigenetic inhibitors

Epigenetic modifications are reversible, in contrast to hereditary mutations. Considering the role that epigenetic marks play in carcinogenesis, there has been a lot of interest in the availability of matching inhibitors. On the other hand, multiple epigenetic events are often needed for the epigenetic control of a gene.

### Targeting DNA methylation

The best defense against aberrant DNA hypermethylation is DNMT blockade. Targeting the methyltransferase enzymes, however, has not yet proven to be selective and has even been known to hypomethylate the entire genome^144^. In mice, complete deletion of DNMT1 causes embryonic death^145^. In fibroblast cells, DNMT1 knockout results in p53-dependent cell death and abnormal expression of 10% of the genes^146^. When male Fischer rats are given DNA methylation inhibitors, cancer occurs. DNA methylation regulation is essential for cell survival and function, but choosing the right medication can be challenging due to its specificity requirements and potential adverse effects^147^.

There are two categories of DNA methylation inhibitors: nonnucleoside analogs and nucleoside analogs. With their altered cytosine ring, nucleoside analogs can be converted into nucleotides and added to freshly created DNA or RNA. Covalent complexes between DNA methyltransferases and the analogs prevent DNA methylation. Now, 5-aza-2′-deoxycytidine (5-Aza-CdR) and 5-aza-cytidine (5-Aza-CR) are the most researched and promising demethylation agents^148^. 5-Aza-CR and zebularine are analogs of ribonucleosides that can phosphorylate to integrate into RNA. Nevertheless, the ribonucleotide reductase route is another way in which they might be integrated into DNA. 5-Azacitidine is an injectable solution used to treat myelodysplastic syndromes (MDSs). It is an analog of cytidine. Demethylation, inactivated gene re-expression, and cell differentiation are all encouraged by it^149^. Fetal abnormalities and decreased male fertility are among the side effects of 5-azacitidine, particularly at high dosages. However, 6-azacytidine, the analog of 5-azacitidine, does not exhibit these effects^150^. The US Food and Drug Administration (FDA) has already approved 5-Aza-CR593 and 5-Aza-CdR594 for the treatment of specific MDS subtypes and chronic myelomonocytic leukemia. Their innate predilection for freshly synthesized DNA causes them to primarily impact cancer cells that are dividing^151^. Clinical trials and preclinical studies investigating their effectiveness in solid tumors are now underway. These nucleoside-like analogs frequently cause genomic instability and mutagenesis risk as adverse effects. Analogs of nonnucleosides can prevent these negative consequences.

Numerous nonnucleoside analogs have been created recently to stop aberrant hypermethylation of DNA. Rather than integrating into DNA, these medications—which are typically tiny chemical inhibitors—directly target catalytic regions. RG108 was created with the intention of demethylating cells by inhibiting the activity of DNMT1, an enzyme that was modeled in three dimensions^152^. Psammaplin is a class of naturally occurring extracts from the sponge *Pseudoceratina purpurea* that exhibits modest cytotoxicity and the ability to inhibit both histone deacetylases and DNA methyltransferases^153^.

Creating antisense oligonucleotides to impede DNMT transcription is an additional tactic. A second-generation phosphorothioate antisense oligodeoxynucleotide, MG98 exhibits no discernible anticancer effects but inhibits the translation effects of DNMT1 mRNA^154^. Preclinical studies and phase I/II clinical trials have looked into it, particularly with solid tumors^155,156^. Notably, 5-Aza-CdR showed superior efficiency in DNA demethylation inhibition in a systemic examination comparing nonnucleoside inhibitors with it^157^. The effectiveness of anti-DNA methylation therapy for different malignancies has been studied in hundreds of clinical trials to date.

### Inhibitors of histone modifications

Histone alterations have been studied in a wider range of disorders than DNA methylation, such as solid tumors, hematological malignancies, and even numerous inflammatory diseases (including viral infections, diabetes, and inflammatory lung diseases). Rather than cytosine methylation or H3K9 demethylation, lysine deacetylation and demethylation of H3K4 may be the main causal events during the process of gene silence^158^. Consequently, histone modification is a prospective target for the therapy of disease since it is crucial to the control of gene expression.

### Antagonists of BETs and HATs

Generally speaking, there are two approaches to stop aberrant histone acetylation: either employing mimetic products of enzyme substrates or changing interactions inside the active sites within HATs. There are now numerous clinical trials examining inhibitors that target BRD proteins, but none that look at inhibitors that target HATs. PCAF, p300, and TIP60 are specifically inhibited by bisubstrate inhibitors. They mimic two HAT substrates: a peptide that resembles the lysine substrate and the cofactor acetyl coenzyme A (Ac-CoA)^159,160^. Nevertheless, because of their peptide makeup and size, they can’t pass through membranes and need a delivery method to help them. Nonpeptide, small molecule inhibitors have been developed as possible medicinal medicines based on HAT inhibitory methods. Natural substances that contain small molecule inhibitors include anacardic acid, curcumin, and garcinol^161,162^. These naturally occurring HAT inhibitors frequently have different targets and are not selective against HATs. Consequently, reports of synthetic and structurally altered substances have been made. Correcting abnormal acetylation during illnesses also requires the appropriate application of HAT agonists. Anacardic acid is the source of CTPB, which preferentially activates p300 to cause gene transcription. Two more anacardic acid-based agonists are TTK21 and SPV106^163^.

Another mechanism that prevents acetylation is binding to BRDs and obstructing the identification of acetylated lysine. Two exemplary BET family inhibitors are JQ1 and I-BET762. Small chemical JQ1 is cell permeable and can attach to BRD4 fusion oncoproteins, like BRD4-NUT, in a competitive manner, which causes cancer cells to differentiate and undergo apoptosis^164^. Similarly, I-BET762 is a synthetic counterpart of BRD4 and a rival of^165^. BET inhibitors include other drugs like MS417, OTX-015, RVX-208, OXFBD, I-BET151, PFI-1, MS436, and XD14, which have been thoroughly documented in other published works^166^.

GlaxoSmithKline (GSK) discovered I-BRD9, a selective inhibitor of BRD9 with over 200-fold selectivity for BRD9 over BRD7 and 700-fold selectivity for BRD9 over members of the BET family. With a higher affinity for the SMARCA4 bromodomain^167^, 619 PFI-3 is a putative inhibitor of both PB1 and SMARCA4. Vangamudi et al. discovered, however, that although PFI-3 did not impede cell growth, the ATPase domain within SMARC4 circumvented the anticancer effects associated with the bromodomain^168^. TRIM24 (tripartite motif-containing protein 24) and BRPF1 both have a bromodomain, which has led to the identification of IACS-9571 as a dual inhibitor. In contrast, researchers found in a recent study that bromodomain inhibitors exclusively targeted the BET family, not other BRDs^169^. Bromosporine is a pan-bromodomain inhibitor with strong cellular action^170^.

### HDAC inhibitors

Since there are several ways to control HDAC activity, there are benefits specific to the categorization of HDAC inhibitors. It was discovered in the 1970s that butyrate caused cancer cells to accumulate acetylated histones, a process that is assumed to be connected to the suppression of deacetylation. Subsequently, it was discovered that trichostatin A (TSA), a natural extract, might cause cancer cell differentiation and apoptosis in addition to inhibiting the activity of partially purified HDACs^171^. Histone deacetylation-inhibiting natural and synthetic substances have been discovered one by one over time. According to a study, the administration of HDAC inhibitors only affects 1%–2% of the genes, but it causes a noticeable and quick decrease in the expression of the c-Myc gene, suggesting that a specific subset of cellular genes was particularly susceptible to the control of histone acetylation^172^.

By up regulating p21, p27, and NF-κB, the combination of two HDAC inhibitors, and TSA, caused melanoma cell growth arrest. MG132 can amplify the effects of TSA^173^. Promising antitumor benefits have been observed when HDAC inhibition is studied in a variety of malignancies^174^. HDAC inhibitors can be categorized into five types based on the properties of their chemical structures: cyclic peptides, hydroxamic acids, benzamides, short-chain fatty acids, and hybrid compounds. Apart from the chemicals classified into the five classes, certain novel synthesized compounds also function as HDAC inhibitors.

Due to the great similarity of the structure and catalytic function of HDACs within each group, early HDAC inhibitors were nonselective. Tubacin, which targets HDAC6 with enhanced tubulin acetylation but not histone acetylation, was the first selective HDAC inhibitor^175^. A particular HDAC8 inhibitor called PCI-34051 can cause caspase-dependent death in T-cell lymphoma cells, although it has no effect on histone acetylation^176,177^.

Notably, second-generation HDACs are presently undergoing clinical trials; some of them have already been approved for the treatment of diseases. These include hydroxamic acids (vorinostat (SAHA), belinostat (PXD101), LAQ824, and panobinostat (LBH589)) and benzamides (entinostat (MS-275), tacedinaline (CI-994), and mocetinostat (MGCD0103). The development of HDAC inhibitors as anticancer medications was expedited by the efficaciousness of romidepsin in phase I clinical trials for cutaneous and peripheral T-cell lymphoma. The US Food and Drug Administration (FDA) first authorized SAHA (vorinostat) in 2006 as an HDAC inhibitor for the treatment of cancer, but only in patients with cutaneous T-cell lymphoma (CTCL). 2009 saw the approval of Romidepsin (Istodax), the second HDAC inhibitor^178^.

### HMT and HDMT inhibitor

By restoring the expression of tumor suppressor genes silenced by abnormal epigenetic alterations, many epigenetic inhibitors—including EPZ-6438 (tazemetostat), a selective inhibitor of the histone methyltransferase EZH2—and RG108, a non-nucleoside inhibitor of DNA methyltransferases (DNMTs)—have shown promising efficacy in preclinical cancer models. The first known selective DOT1L inhibitor, EPZ004777, preferentially kills cells with MLL translocation^142^. However, a second version of EPZ004777, known as EPZ-5767, was created with a cyclobutyl ring in place of the ribose moiety due to its poor pharmacokinetic qualities^179^. When treating ALL with MLL translocation, EPZ-5767 exhibits synergistic benefits when combined with cytarabine, daunorubicin, and the DNMT inhibitor azacitidine. Despite its limited oral bioavailability, EPZ-5767 has been studied in clinical studies for the treatment of leukemia with MLL rearrangement^180^.

Competitive inhibitors are an additional class of inhibitors. KMTs’ methyl moiety is the result of SAM. EI1, a tiny molecule inhibitor of EZH2, competes with SAM and binds directly to EZH2 to limit EZH2 activity^181^. Two more SAM competitive inhibitors that have been studied in clinical studies are GSK343 and GSK126. Without evident effects on the growth of wild-type cells, EPZ005687, a strong inhibitor of EZH2, drastically lowers H3K27 methylation in lymphoma cells with point mutations at the Tyr641 and Ala677 residues of EZH2^182,183^. The next drug to be developed was EPZ-6438, which had better oral bioavailability and comparable effects.654 CPI-1205 is a new member of the pyridone family and an EZH2 inhibitor^184^.

Because tranylcypromine (TCP) inhibits the activity of monoamine oxidase (MAO), it is a medication licensed for the treatment of depression. There are numerous structural similarities between MAOs and Lysine-Specific Demethylase (LSD) enzymes. Thus, targeting MAO is primarily responsible for the side effects of TCP as an HDMT inhibitor, which include orthostatic hypotension, vertigo, and sleepiness^185^. TCP treatment increases tumor cell differentiation and death in MLL-AF9 leukemia^186^.

The process of epigenetic regulation during carcinogenesis is intricate and requires several steps. As a result, it appears beneficial to combine two or more medicines that target different epigenetic events. Together, these factors suppress the expression of genes that encourage tumor growth and encourage the re-expression of tumor suppressor genes. Small molecules like 4SC-202 have two different actions: they can inhibit LSD1 and HDAC1/2/3 with low micromolar potency. Clinical research is being done on this medication. Typically producing transcriptional suppression, LSD1 (KDM1A) is a well-characterized flavin-dependent histone demethylase that preferentially demethylates mono- and dimethylated lysine 4 on histone H3 (H3K4me1/2). LSD1 can, however, demethylate H3K9me1/2 to promote gene activation when coupled with particular cofactors, highlighting its context-dependent dual regulatory function. Essential for stem cell maintenance, differentiation, and oncogenesis, LSD1 makes an appealing therapeutic target in several cancers^187^.

Over the last twenty years, the clinical translation of epigenetic research has advanced quickly, leading to the development and approval of various epigenetic-targeted therapies (epidrugs) for various cancers. Recent developments in epigenetic therapies have led to the clinical evaluation and approval of several “epidrugs” targeting DNA methylation pathways and histone changes. While histone deacetylase (HDAC) inhibitors like Vorinostat (licensed for cutaneous T-cell lymphoma) have demonstrated efficacy, their clinical use is often limited by off-target effects, including fatigue, thrombocytopenia, and gastrointestinal toxicities^188^. Similarly, DNA methyltransferase (DNMT) inhibitors like Azacitidine and Decitabine have shown survival benefits in acute myeloid leukemia (AML) and myelodysplastic syndromes (MDS); yet, hematological toxicities and limited efficacy in fast proliferating AML cells remain challenges^189,190^. Though resistance and secondary cancers have developed, the 2020 approval of Tazemetostat, an EZH2 inhibitor, for relapsed/refractory follicular lymphoma and advanced epithelioid sarcoma marked a significant development in the field by offering a targeted approach against histone methyltransferase dysregulation^191^. Often researched in combination treatments to overcome natural resistance mechanisms, more HDAC inhibitors, including Belinostat (approved for peripheral T-cell lymphoma) and experimental drugs like Entinostat (showing promise in breast cancer and non-small cell lung cancer), have expanded the therapeutic landscape 173,174. Despite these developments, Guadecitabine and other next-generation DNMT inhibitors failed to meet survival goals in Phase III AML studies, hence stressing the complexity of epigenetic reprogramming and the need for better biomarker-driven patient selection^192^. Table 2 briefly describes these important clinical trials, emphasizing their goals, cancer types, findings, and limitations to create a more transparent translational framework for epigenetic therapies in oncology. It also provides a list of key clinical trials of epigenetic therapies across cancer types.

**Table 2:** Key clinical trials of epigenetic therapies across cancer types

Drug	Target	Cancer Type(s)	Clinical Trial Phase	Outcomes	Limitations	References
**Vorinostat (SAHA)**	HDAC inhibitor	Cutaneous T-cell lymphoma (CTCL), solid tumors	Approved (CTCL); Ongoing trials (solid tumors)	FDA-approved for CTCL (2006); partial responses in solid tumors, but limited durable efficacy	Off-target effects: fatigue, thrombocytopenia, gastrointestinal toxicities	Duvic et al.^205^
**Azacitidine (Vidaza)**	DNMT inhibitor	Myelodysplastic syndromes (MDS), AML	Approved (MDS); Ongoing (AML)	Improved overall survival in MDS; modest benefit in elderly AML patients	Myelosuppression; infection risk; limited effect in rapidly proliferating AML	Fenaux et al.^189^
**Decitabine**	DNMT inhibitor	MDS, AML	Approved (MDS); Ongoing (AML)	Improved response rates in older AML; approved for MDS	Hematologic toxicities (neutropenia, anemia); infection	Garcia-Manero et al.^206^
**Tazemetostat (Tazverik)**	EZH2 inhibitor	Follicular lymphoma, epithelioid sarcoma	Approved (2020)	Durable objective responses in relapsed/refractory follicular lymphoma and advanced epithelioid sarcoma	Resistance emergence; secondary malignancies; fatigue, nausea	Hoy, 2020^191^
**Belinostat**	HDAC inhibitor	Peripheral T-cell lymphoma (PTCL)	Approved (2014)	Accelerated approval for PTCL; ~26% overall response rate	Hematologic toxicity; infections; fatigue	O’Connor et al.^207^
**Entinostat**	HDAC inhibitor	Breast cancer, NSCLC	Phase III (ongoing)	Shown to sensitize tumors to immune checkpoint inhibitors; improvement in PFS in combination with endocrine therapy	Side effects include neutropenia, fatigue; requires combination for maximal efficacy	Yardley et al.^208^
**CC-486 (oral Azacitidine)**	DNMT inhibitor	AML, MDS	Approved (2020)	Maintenance therapy post-AML remission; prolongs survival	Gastrointestinal events; cytopenias	Wei et al.^209^
**Valemetostat**	Dual EZH1/2 inhibitor	Adult T-cell leukemia/lymphoma (ATLL)	Phase II (completed)	Promising efficacy in relapsed/refractory ATLL; ORR ~ 48%	Potential for epigenetic reprogramming resistance; hematologic adverse events	Zinzani, et al.^210^
**Guadecitabine**	DNMT inhibitor (next-gen)	AML, MDS	Phase III (discontinued)	Failed to significantly improve OS in Phase III AML trial	Lack of survival benefit; adverse effects similar to earlier DNMT inhibitors	Fenaux et al.^211^

Summary of selected epigenetic-targeted drugs (epidrugs), including their molecular targets, associated cancer types, clinical trial phases, primary outcomes, and noted limitations. This overview highlights the translational impact and current challenges of epigenetic therapies in oncology.

## Conclusion

In both cancer and regular cell activity, epigenetic changes are considered vital. In cancer, these changes influence several processes including signal transduction, the regulation of oncogenes and tumor suppressor genes, cell proliferation, invasion, and metastasis. Though its use in solid tumors is limited, emphasizing the need for better mechanical understanding and improved treatment strategies, epigenetic therapy and its clinical effectiveness in hematological cancers provide compelling reasons. Finding and characterizing epigenetic abnormalities specific to cancer kinds vital for tumor sustenance and progression is really essential if the field is to be advanced. This will help to speed the development of more focused therapeutic drugs. Still, additional challenges call for remedy. Epigenetic changes are not exclusive to cancerous cells; they are also required for proper cellular balance. Therapeutic targeting therefore raises significant questions about selectivity, off-target effects, unintentional gene silencing, and systemic toxicity in healthy tissues. The translation of epigenetic therapies into broader clinical use presents a major difficulty in striking a balance between effectiveness and safety. Moreover, the combination of epigenetic therapy with other modalities, particularly immunotherapy, is a hopeful but under-researched field. Studies done lately show that epigenetic drugs can change the immunological environment of tumors, hence possibly enhancing the efficacy of immune checkpoint blockers. Systematic studies are required to define the mechanisms underlying this synergy and to identify ideal combination regimens. Ultimately, while epigenome-targeted therapy is a creative and hopeful approach for cancer treatment, especially when used with immunotherapies, a proactive attitude should include careful work to handle issues relating to specificity, safety, and resistance mechanisms. Though epigenetic therapy shows promise, problems like on-target toxicity—for example, thrombocytopenia linked to DNMT inhibitors—demand exact approaches. Unlocking the full therapeutic potential of epigenetic control in cancer will depend on addressing these research gaps through mechanistic studies and systematic medication design.

## Data Availability

No datasets were generated or analyzed during the current study.
